# Breast cancer survival in sub‐Saharan Africa by age, stage at diagnosis and human development index: A population‐based registry study

**DOI:** 10.1002/ijc.32406

**Published:** 2019-06-14

**Authors:** Walburga Y. Joko‐Fru, Adalberto Miranda‐Filho, Isabelle Soerjomataram, Marcel Egue, Marie‐Therese Akele‐Akpo, Guy N'da, Mathewos Assefa, Nathan Buziba, Anne Korir, Bakarou Kamate, Cheick Traore, Shyam Manraj, Cesaltina Lorenzoni, Carla Carrilho, Rolf Hansen, Anne Finesse, Ntuthu Somdyala, Henry Wabinga, Tatenda Chingonzoh, Margaret Borok, Eric Chokunonga, Biying Liu, Eva Kantelhardt, Paul McGale, Donald M. Parkin

**Affiliations:** ^1^ The African Cancer Registry Network INCTR African Registry Programme Oxford United Kingdom; ^2^ Nuffield Department of Population Health University of Oxford Oxford United Kingdom; ^3^ Section on Cancer Surveillance International Agency for Research on Cancer Lyon France; ^4^ Cotonou Cancer Registry Cotonou Benin; ^5^ Abidjan Cancer Registry Abidjan Cote d'Ivoire; ^6^ Addis Ababa City Cancer Registry Addis Ababa Ethiopia; ^7^ Eldoret Cancer Registry Eldoret Kenya; ^8^ Nairobi Cancer Registry Nairobi Kenya; ^9^ Bamako Cancer Registry Bamako Mali; ^10^ Mauritius National Cancer Registry Port Louis Mauritius; ^11^ Maputo Cancer Registry Maputo Mozambique; ^12^ Namibian Cancer Registry Windhoek Namibia; ^13^ Seychelles National Cancer Registry Victoria Seychelles; ^14^ Eastern Cape Cancer Registry Tygerberg South Africa; ^15^ Kampala Cancer Registry and Department of Pathology, School of Biomedical Sciences, College of Health Sciences Makerere University Kampala Uganda; ^16^ Bulawayo Cancer Registry Bulawayo Zimbabwe; ^17^ Zimbabwe National Cancer Registry Harare Zimbabwe; ^18^ Department of Gynaecology and Institute of Medical Epidemiology, Biostatistics and Informatics Martin‐Luther University Halle‐Wittenberg Halle Germany; ^19^ Clinical Trial Service Unit, Nuffield Department of Population Health University of Oxford Oxford United Kingdom

**Keywords:** breast cancer, stage, human development index, survival, Africa

## Abstract

Breast cancer is the leading cancer diagnosis and second most common cause of cancer deaths in sub‐Saharan Africa (SSA). Yet, there are few population‐level survival data from Africa and none on the survival differences by stage at diagnosis. Here, we estimate breast cancer survival within SSA by area, stage and country‐level human development index (HDI). We obtained data on a random sample of 2,588 breast cancer incident cases, diagnosed in 2008–2015 from 14 population‐based cancer registries in 12 countries (Benin, Cote d'Ivoire, Ethiopia, Kenya, Mali, Mauritius, Mozambique, Namibia, Seychelles, South Africa, Uganda and Zimbabwe) through the African Cancer Registry Network. Of these, 2,311 were included for survival analyses. The 1‐, 3‐ and 5‐year observed and relative survival (RS) were estimated by registry, stage and country‐level HDI. We equally estimated the excess hazards adjusting for potential confounders. Among patients with known stage, 64.9% were diagnosed in late stages, with 18.4% being metastatic at diagnosis. The RS varied by registry, ranging from 21.6%(8.2–39.8) at Year 3 in Bulawayo to 84.5% (70.6–93.5) in Namibia. Patients diagnosed at early stages had a 3‐year RS of 78% (71.6–83.3) in contrast to 40.3% (34.9–45.7) at advanced stages (III and IV). The overall RS at Year 1 was 86.1% (84.4–87.6), 65.8% (63.5–68.1) at Year 3 and 59.0% (56.3–61.6) at Year 5. Age at diagnosis was not independently associated with increased mortality risk after adjusting for the effect of stage and country‐level HDI. In conclusion, downstaging breast cancer at diagnosis and improving access to quality care could be pivotal in improving breast cancer survival outcomes in Africa.

## Abstract 2

**What's new?**

Breast cancer is the leading cancer diagnosis and second most common cause of cancer deaths in sub‐Saharan Africa (SSA). Yet there have been few studies in this region on survival differences by stage at diagnosis. Here, the authors used cancer registry data to analyze differences in breast‐cancer survival by age, stage at diagnosis and country‐level human development index. They conclude that downstaging and improving access to quality care could be pivotal in improving breast‐cancer survival outcomes in Africa.

**Abbreviations**
AFCRN: African Cancer Registry Network
AJCC: American Joint Committee on Cancer
ASRS: age‐standardized relative survival
CI: confidence interval
DCO: death certificate only
GNI: Gross National Income
HDI: human development index
IARC: International Agency for Research on Cancer
ICD‐10: International Classification of Disease 10th revision
ICSS: International Cancer Survival Standard
KM: Kaplan–Meier
LFU: loss to follow‐up
LMIC: low‐ and middle‐income countries
MIR: mortality to incidence ratio
MV: microscopically verified
PBCR: population‐based cancer registry
RS: relative survival
SSA: sub‐Saharan Africa
TNM: tumor node metastasis
WHO: World Health Organization

## Introduction

Breast cancer is the leading cause of cancer morbidity and the second most important cause of mortality from cancer in sub‐Saharan Africa (SSA).^1^ Although the incidence rates in Africa are the lowest in the world, its mortality rates are highest, reflecting the poorer survival outcomes. Survival statistics have been used as an important tool for monitoring progress in cancer diagnosis and treatment.^2^ However, there are relatively few population‐based African cancer registries represented in international collaborative studies. In the SURVCAN‐2 studies,^3^ three SSA countries were represented, with an estimated 5‐year age‐standardized relative survival (RS) for cases diagnosed from 1993 to 1997 at 12.5% in The Gambia, 42.9% in Harare and 45.8% in Kampala. In the CONCORD‐3 studies, four African countries south of the Sahara were represented; this study reported a high level of variability in the age‐standardized 5‐year net survival from breast cancer, with estimates ranging from 0% in Mali to 97.5% in Ibadan, Nigeria during the 2010–2014 period.^4^

Assessment of the progress that has been made in breast cancer diagnosis and treatment in SSA will be hampered if survival estimates are not accurately measured. Previous studies have shown the importance of active follow‐up in countries where mortality linkage is poor, and our study makes use of actual data generated by population‐based cancer registries (PBCR), enhanced by active follow‐up and detailed medical record studies to obtain high‐quality datasets with comprehensive clinical data.

The African Cancer Registry Network (AFCRN), in collaboration with the International Agency for Research on Cancer (IARC) and the individual registries, provided a unified framework for cancer registration and monitoring of survival across 14 PBCR from 12 SSA countries. In this article, we present estimates of breast cancer population‐based survival in greater depth than previously published, investigating the effects of age and stage at diagnosis on survival at 1, 3 and 5 years after diagnosis among women in SSA.

## Methods

### Study population

Data were obtained from the AFCRN. We included malignant breast cancer cases (ICD‐10: C50) diagnosed among black African females aged 15 and above. A random sample of incident cases diagnosed in 2008–2015 was selected from each registry. The number of cases sampled per registry was determined by the practical feasibility of obtaining follow‐up information. Where patient follow‐up was passive (see below), a larger number of cases could be included, when active methods were used the sample was smaller and the fraction was then a function of the total incident cases in the period concerned. None of the cases had been previously diagnosed with a breast cancer; we did not exclude breast cancer cases who had a previous cancer at a different site, although such cases are rather rare in an African setting. The follow‐up time was measured from the date of incidence until the date of last contact, the date of death or until the end of the study (December 31, 2017), whichever occurred first.

### Vital status

This was obtained by active methods for all but one registry (Mauritius). In active follow‐up, clinical records are traced and the patient's vital status at the closing date recorded. Cases whose vital status could not be confirmed at the end of this procedure were called when a mobile number was registered in the registry record. When no further information could be obtained, home visits were made by the registry staff. Patients whose vital status (alive/dead) could not be ascertained by the closing date of the study were censored “alive.” In Mauritius, passive follow‐up was done to ascertain the vital status of patients; this involves linkage of the list of registered cases with the population death records held in the vital statistics office. Patients not found to have died are assumed to be still alive.

### Data analyses

For each registry, we calculated the sampling fraction, the mean age at diagnosis and proportion of cases with microscopic verification. We excluded cases diagnosed based on a death certificate only (DCO), with no follow‐up information and with incoherent follow‐up dates. We used the semi‐complete approach,^5^ which uses the survival probabilities of patients with complete follow‐up (diagnosed 5 years prior to the closing date) and the survival probabilities of patients diagnosed more recently. We present Kaplan–Meier (KM) survival curves and estimate the observed and Ederer II RS at 1, 3 and 5 years after diagnosis using the “strs”^6^ command in STATA 14. The RS is the ratio of the “observed” survival in the study population to the “expected” survival.^7^ The expected survival derived from country‐specific lifetables is the survival experience of the general population of the same age, sex and period. As such, differences in background mortalities of cases are taken into account. Age standardization was done using the International Cancer Survival Standard (ICSS)–1^8^, for cancers whose incidence increases with age.

### Lifetables

Abridged life tables by sex and country were obtained from the WHO lifetable database.^9^ Mortality probability were expanded using a Poisson regression model to obtain a complete lifetable by 1‐year age group and period of diagnosis. Additional information on modeling the lifetables is found in the Supporting Information.

### Stage at diagnosis

Whenever available, information on stage had been abstracted at time of registration by the registrars. Stage was categorized using the tumor–node–metastasis (TNM) system^10^ in all registries. From individual categories of tumor, node and metastasis, stage was classified into four stages (I–IV) using the anatomic stage groupings of the American Joint Cancer Committee (AJCC) TNM8 classification for breast cancer.^11^ For registries with no individual T, N and M data, we used the available summary stage information. Stages I and II were grouped as “Early Stage” and Stages III and IV as “Late Stage.” Records with no information on stage were grouped into a separate category referred to as “Missing Stage.”

### Assessing loss to follow‐up

The proportion of loss to follow‐up (LFU) was assessed at 1, 3 and 5 years after diagnosis. Using a Cox model with patients LFU as the outcome, we evaluated whether LFU was random or if it was associated with either age or stage at diagnosis.

### Human development index classification

The human development index (HDI) is a composite measure developed by the United Nations Development Programme that aims at assessing the level of development of countries.^12^ It has three main components: life expectancy at birth, the educational attainment of citizens and the Gross National Income (GNI) *per capita*. We used the HDI 2015^13^ classification to categorize the included countries and compared survival within SSA by HDI.

### Modeling excess hazards

We modeled the excess hazard of death in a RS framework for patients with breast cancer as a function of age at diagnosis, stage and country HDI using a Poisson regression model.^6^ We split time into monthly intervals and made use of restricted cubic splines. We fitted an interaction term between age at diagnosis and stage at diagnosis, to assess if the effect of age is constant within these categories. We used the likelihood ratio test to compare the main model with the models that include the interaction parameter.

## Results

In total, there were 2,558 randomly selected cases from 12 countries, representing 30% of the total female breast cancers diagnosed in 14 individual PBCR within the study period. These registries had national coverage in Mauritius, Namibia and Seychelles; covered an urban area for all the other registries except for the Eastern Cape registry which covers a rural area. Only black African females were included—cases among women of European, Asian or mixed‐race origin were excluded. We also excluded cases without any follow‐up information after the date of diagnosis, or with incoherent dates, and this proportion ranged from 0% in Cotonou, Benin to 31% in Eldoret, Kenya (Table 1). Finally, 2,311 cases (90.3%) were included in our study. Of the total number of incident breast cancer cases, the proportion included per registry ranged from 7.9% in Bamako, Mali to 100% in Eastern Cape, South Africa. The proportion of cases microscopically verified (MV%) ranged from 54.7% in Kampala, Uganda to 100% in Maputo, Mozambique and Namibia.

**Table 1:** Total number of breast cancer diagnosis, included and excluded cases, and data quality indicator by population‐based cancer registry

Country	HDI 2015	Registry	Period of diagnosis	Total number of breast cancer cases during study period	No. (%) of DCO during study period excluded	Number of randomly sampled cases for survival study	Sampling fraction (%)	Histologically verified (%)	Included for survival analyses *n* (%)	No. excluded (%)
Benin	Low	Cotonou	2013–2014	132	0 (0.0)	91	68.9	79.1	91 (100)	0
Cote d'Ivoire	Low	Abidjan	2012–2013	531	23 (4.3%)	242	47.6	81.8	209 (86.4)	33 (13.6)
Ethiopia	Low	Addis	2012	437	0 (0.0)	418	95.7	94.7	389 (93.1)	29 (6.9)
Kenya	Medium	Eldoret	2009–2013	307	15 (4.9%)	113	38.7	97.4	78 (69.0)	35 (31.0)
Kenya	Medium	Nairobi	2009–2013	1,544	80 (5.1%)	148	10.1	91.9	141 (95.3)	7 (4.7)
Mali	Low	Bamako	2012–2013	639	5 (0.8%)	50	7.9	96	48 (96.0)	2 (4.0)
Mauritius	High	Mauritius	2005–2009	1,616	5 (0.3%)	500	31.0	95.4	491 (98.2)	9 (1.8)
Mozambique	Low	Maputo	2015	81	14 (17.8%)	43	64.2	100	42 (97.7)	1 (2.3)
Namibia	Medium	Namibia	2012–2013	454	0 (0.0)	75	16.5	100	64 (85.3)	11 (14.7)
Seychelles	High	Seychelles	2008–2013	124	1 (0.8%)	111	90.2	93.7	105 (94.6)	6 (5.4)
South Africa	Medium	Eastern Cape	2008–2013	369	0 (0.0)	369	100	87	313 (84.8)	56 (15.2)
Uganda	Low	Kyadondo (Kampala)	2009–2013	645	19 (2.9%)	150	24	54.7	112 (74.7)	38 (25.3)
Zimbabwe	Low	Bulawayo	2012–2013	167	16 (9.6%)	57	37.8	89.5	54 (94.7)	3 (5.3)
	Low	Harare	2009–2013	725	50 (6.7%)	191	28.3	93.7	174 (91.1)	17 (8.9)
Total cases						2,558		91.3	2,311 (90.3)	247 (9.7)

Abbreviations: DCO, death certificate only; HDI, human development index.

Of the cases included for survival analyses (Table 2), the mean age at diagnosis ranged from 45.8 years in Addis Ababa, Ethiopia to 59.6 in Seychelles, with a median duration of follow‐up ranging from 8.5 months in Cotonou, Benin to 5.4 years in Mauritius. Almost half of the patients (47.4%) were diagnosed under the age of 50. The distribution of cases by broad age group in each registry is shown in Supporting Information Figure S1.

**Table 2:** Patient's characteristics: Mean age at diagnosis, median years of follow‐up and observed (all‐cause) survival

Country, Registry	Number of cases included	Mean age at diagnosis	Year 1				Years 2 and 3				Years 4 and 5		
			No. of deaths (%)	LFU	% LFU	1‐year Observed Survival (in %)	No. of deaths (%)	LFU	%LFU	3‐year observed survival (in %)	No. of deaths (%)	5‐year observed survival (in %)	Median follow‐up (years)
Benin, Cotonou	91	46.8	10 (10)	44	45	81.8	12 (13)	5	6	53.0	**–**	**–**	0.7
Cote d'Ivoire, Abidjan	209	47.3	27 (13)	39	19	85.1	55 (26)	6	3	51.2	**–**	**–**	2.3
Ethiopia, Addis	389	45.8	33 (9)	46	12	90.7	91 (23)	7	2	63.6	**–**	**–**	3.3
Kenya, Eldoret^1^	78	47.2	10 (13)	22	28	83.6	17 (22)	7	9	49.5	3 (4)	40.1	1.2
Kenya, Nairobi^1^	141	48.4	7 (5)	38	27	94.4	17 (12)	17	12	75.8	8 (6)	64.0	2.5
Mali, Bamako	48	46.2	12 (25)	8	17	71.4	10 (21)	3	6	44.1	**–**	–	1.2
Mauritius^1^	491	55.9	51 (10)	0	0	89.5	58 (12)	0	0	77.6	20 (4)	73.2	5.4
Mozambique, Maputo	42	50.3	8 (19)	12	28	75.8	**–**	**–**	**–**	**–**	**–**	**–**	1.1
Namibia^1^	64	53.3	3 (5)	5	8	95.0	9 (14)	3	5	79.1	4 (6)	70.4	4.5
SA, Eastern Cape^1^	313	55.8	70 (22)	61	20	74.5	66 (21)	29	9	44.9	19 (6)	33.3	1.3
Seychelles^1^	105	59.6	14 (13)	0	0	86.7	11 (11)	1	1	76.2	13 (12)	61.2	4.2
Uganda, Kyadondo^1^	112	47.4	25 (22)	30	27	74.1	29 (26)	8	7	31.5	10 (9)	11.1	1.0
Zimbabwe, Bulawayo	54	57.1	17 (32)	17	32	61.0	12 (22)	5	9	19.3	**–**	**–**	0.8
Zimbabwe, Harare^1^	174	52.4	45 (26)	0	0	74.0	39 (22)	1	0.6	51.3	12 (7)	43.5	3.3

1 Registries with a potential follow‐up time of 5 years (or more).

Abbreviation: LFU, lost to follow‐up.

We obtained information on stage at diagnosis for all registries except for Mauritius. Of the 13 registries which submitted data on stage at diagnosis, stage was known for 47% of patients (892 women). Patients from Seychelles and Namibia (of high and medium HDI respectively) had the greatest proportions of early‐stage diagnosis (Supporting Information Fig. S2). Among patients with known stage, 64.9% were diagnosed at a late stage (Stages III and IV) with 18.4% being metastatic at diagnosis (Stage IV).

### Assessing LFU

The proportion of cases LFU was generally highest in the first year following diagnosis (Table 2). However, among those for whom we had information on stage, LFU at Year 1 was not related to stage or age at diagnosis for all registries, when assessed in a Cox model with LFU as outcome and adjusted for the effect of age and stage at diagnosis. When LFU at Year 3 was assessed, LFU was nondifferential by age and stage in all registries except for Abidjan, where patients diagnosed at late stage had a greater risk of being LFU, as did patients aged 45–54.

Of the 14 registries, eight registries had a cohort of patients with a potential for complete 5‐year follow‐up by the closing date. The proportion of cases with 5‐year complete FU ranged from 56% in Eldoret to 100% in Mauritius (Supporting Information Table S1). For the other registries, we evaluated survival only at 1 and 3 years after diagnosis.

### Survival statistics for all ages by registry

**Figure 1:**
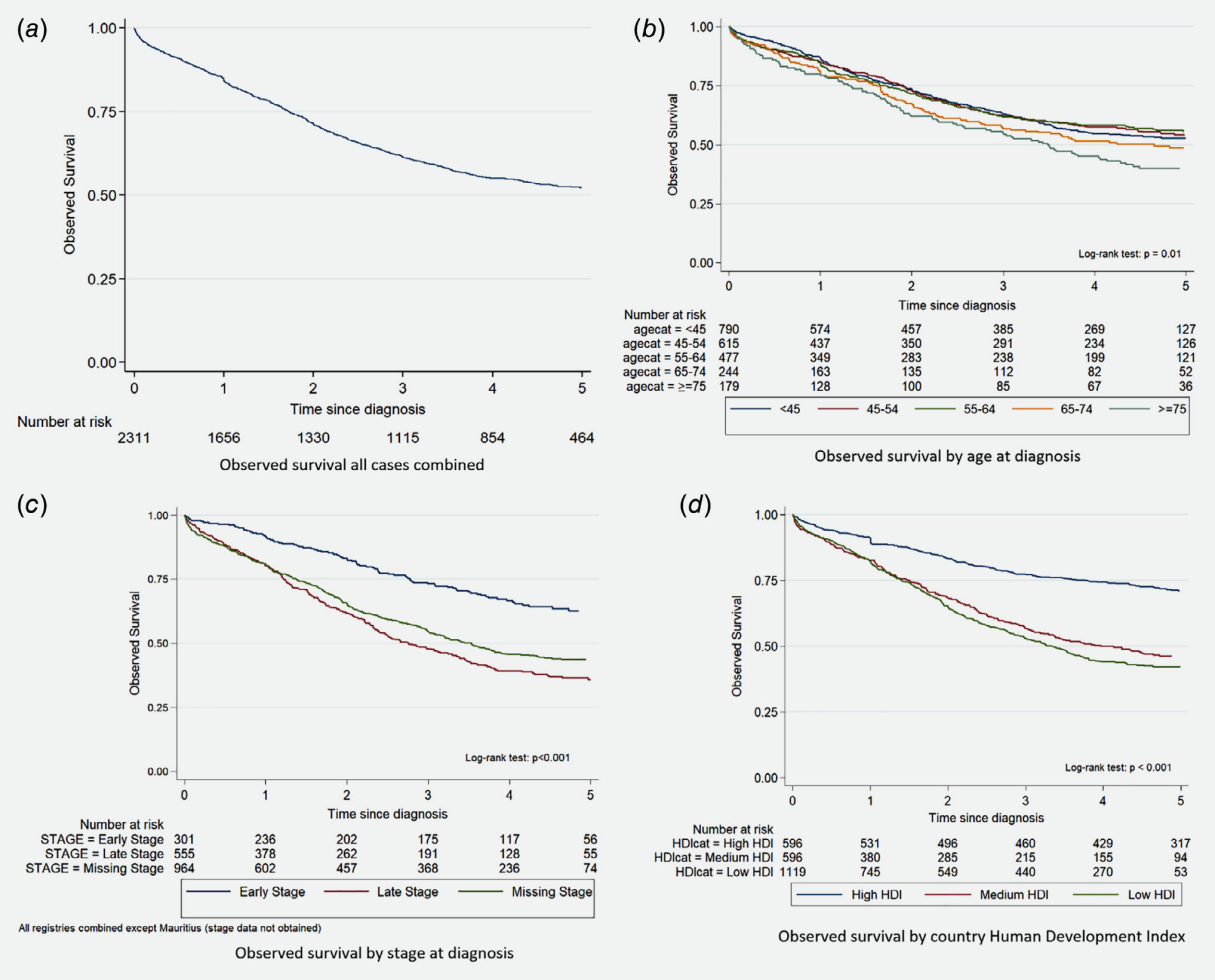
Overall Kaplan–Meier survival for all registries combined (*a*), by age (*b*), stage (*c*) and country level Human Development Index (*d*).

The overall all‐cause Kaplan–Meier (KM) survival was 84.1% (82.5–85.6) at Year 1, 61.4% (59.1–63.5) at Year 3 and 52.3% (49.9–54.6) at Year 5 (Fig. 1). The all‐cause KM survival was lowest in the oldest age group (Fig. 1). Supporting Information Figure S3 shows the KM survival by registry, the 5‐year all‐cause survival was lowest in Kampala and highest in Mauritius.

**Figure 2:**
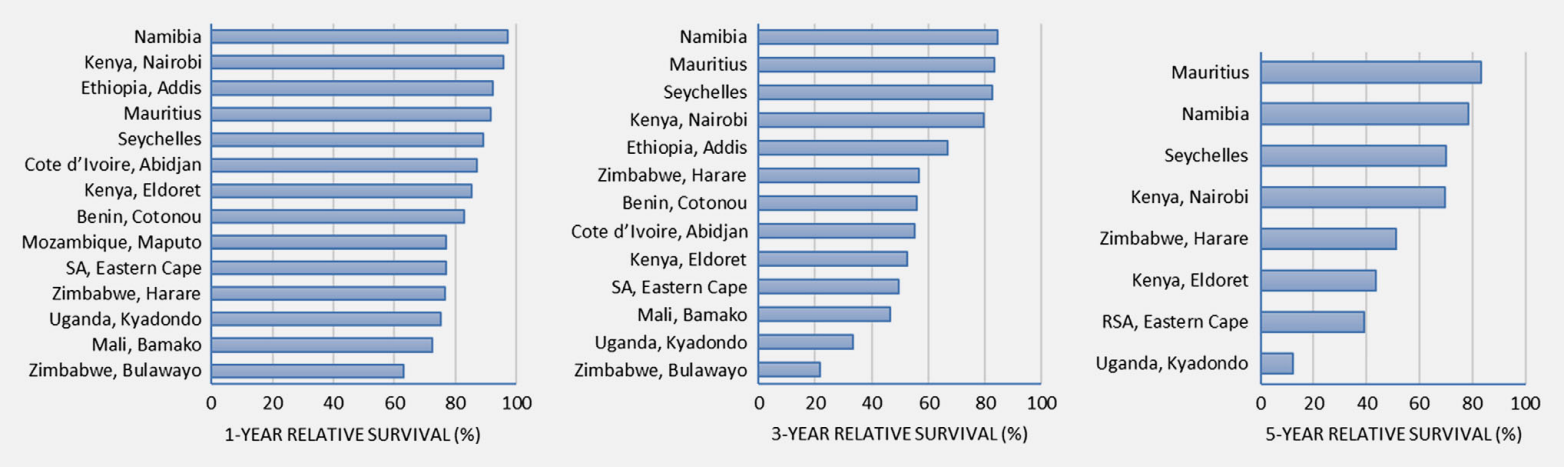
Relative survival (RS) from breast cancer at 1, 3 and 5 years after diagnosis, by registry. [Color figure can be viewed at http://wileyonlinelibrary.com]

The RS at Year 1 was highest in countries of medium or high HDI ranging from 97.1% in Namibia to 63.0% in Bulawayo, Zimbabwe (Fig. 2). Similarly, at 5 years after diagnosis, RS was highest in Mauritius at 83.2% and lowest in Kyadondo, Uganda at 12.1% (Fig. 2). The overall RS at Year 1 for the entire cohort was 86.1% (84.4–87.6), 65.8% (63.5–68.1) at Year 3 and 59.0% (56.3–61.6) at Year 5.

The corresponding overall age‐standardized relative survival (ASRS) for female breast cancer patients in our cohort was 86.3% (83.4–88.8) in Year 1, 70% (65.6–74.0) in Year 3 and 66.3% (60.4–71.5) in Year 5. We observe disparities within SSA in the 5‐year ASRS by registry, ranging from 5.3% (1.9–11.3) in Kyadondo, Uganda to 93.7% (75.5–98.5) in Mauritius (Supporting Information Table S2). We equally observe survival differences within the same country, with better survival rates seen for patients diagnosed in the capital cities of Zimbabwe (Harare) and Kenya (Nairobi).

### Survival by age at diagnosis and registry

There were no systematic trends in RS with age. Women younger than 45 at diagnosis had lower survival point estimates at Year 3 compared to women in the 55–64 age group in Seychelles, Cotonou, Bamako, Namibia, although with wide and generally overlapping confidence intervals (CIs; Supporting Information Table S2).

### Survival by stage at diagnosis

Survival differed by stage at diagnosis, patients diagnosed at an early stage had a 62.5% (55.6–68.6) 5‐year KM survival probability and those diagnosed at a late stage at 35.8% (30.9–40.7) for all registries combined (with the exception of Mauritius; log‐rank test *p* < 0.001; Fig. 1). Supporting Information Table S3 shows the RS by stage at diagnosis for each registry, and we observe differences in survival for patients of the same stage by registry.

### Survival by country‐level HDI

**Figure 3:**
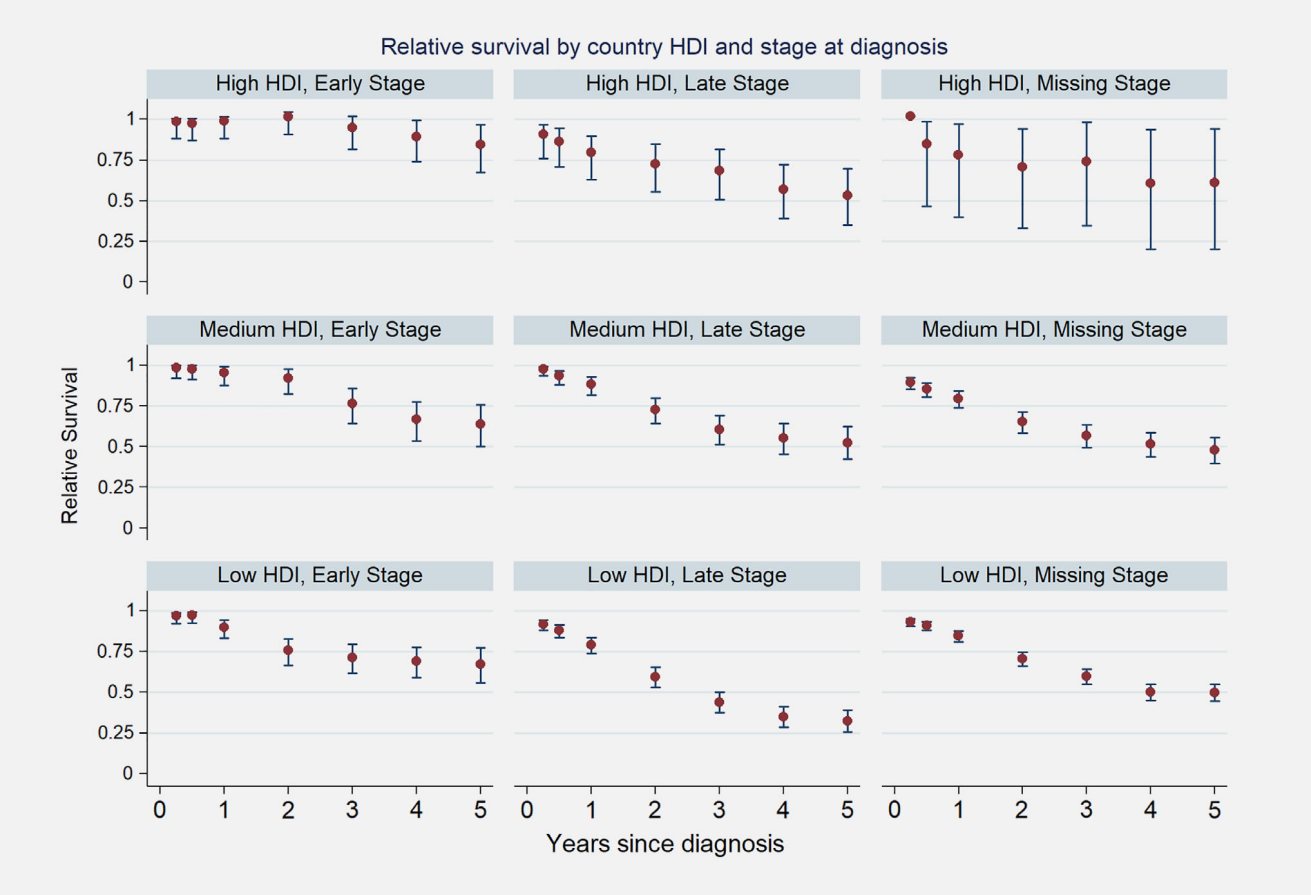
Relative survival by country‐level Human Development Index (HDI) and stage at diagnosis. [Color figure can be viewed at http://wileyonlinelibrary.com]

The survival experience of patients diagnosed in countries with a high HDI was better than for patients in countries with low and middle HDI (Fig. 1). Cases from countries with a high HDI were diagnosed on average at age 56.5 years while cases from a low HDI country at age 48. Figure 3 shows differences of the RS by HDI, even after categorization by stage. For patients diagnosed at Stages III and IV, patients diagnosed in countries with a high HDI had a 5‐year RS estimated at 53.4% (35.0–69.5) while patients in countries with low HDI at 31.9% (25.4–38.5). If diagnosed early, patients in high HDI country have a 5‐year RS estimated at 84.7% (67.4–96.9) while for patients from low HDI countries it 67.1% (55.4–77.0).

### Excess hazard ratio; incorporating the effect of age, stage and country HDI

The excess hazard was 2.5 (1.8–3.3) times higher for patients diagnosed at a late stage compared to patients diagnosed early, even after controlling for the effect of the country HDI (Table 3). The country HDI was equally independently associated with an increased hazard of death, with patients diagnosed in a country with either a medium or low HDI having a hazard of death twice that in a country of high HDI, even after controlling for the stage at diagnosis (Table 3). However, age at diagnosis was not an independent predictor of higher excess hazards after controlling for the effect of stage and country HDI in our model. There was no evidence of an interaction between age and stage at diagnosis.

**Table 3:** Breast cancer excess mortality hazard by stage, country HDI and age at diagnosis

		Univariable analysis			Multivariable adjusted^1^ model		
Prognostic factors	Number of cases	Hazard ratio	95% CI	*p* value	Hazard ratio	95% CI	*p* value
Age at diagnosis (years)							
<45	790	1 (Ref)			1 (Ref)		
45–54	615	1.0	0.8–1.2	0.83	1.0	0.8–1.2	0.1
55–64	477	1.1	0.9–1.3	0.61	1.2	0.9–1.4	0.2
65–74	244	0.9	0.7–1.3	0.70	1.1	0.8–1.5	0.33
75+	179	0.9	0.6–1.4	0.70	0.9	0.6–1.5	0.57
Country‐level HDI							
High HDI	596	1 (Ref)			1 (Ref)		
Medium HDI	596	2.3	1.4–3.8	0.001	1.9	1.2–3.1	0.01
Low HDI	1,119	2.8	1.7–4.5	<0.001	2.3	1.4–3.7	0.001
Stage at diagnosis							
Early stage	301	1 (Ref)			1 (Ref)		
Late stage	555	2.7	2.0–3.6	<0.001	2.5	1.8–3.3	<0.001
Unknown stage	962	2.2	1.6–2.9	<0.001	1.9	1.4–2.5	<0.001

1 Adjusted for stage at diagnosis, country‐level human development index (HDI) and age at diagnosis.

Abbreviation: 95% CI, 95% confidence interval.

## Discussion

Population‐level survival statistics from high‐income countries are widely available,^14^, ^15^, ^16^ but these are very sparse from SSA. Results from individual cancer registries have been published previously,^17^, ^18^ and limited data have been published in previous international compilations.^3^, ^4^ To the best of our knowledge, this is the first study that compares survival differences within SSA by age and stage at diagnosis using population‐level data. It also includes the largest number of population‐based cancer registries (PBCR) in a single comparative study on survival.

We observed a wide variation in survival from breast cancer within SSA, with the lowest survival observed in patients diagnosed at a late stage and in countries with a low HDI. For all cases combined, the 5‐year ASRS was 66.3% (60.4–71.5), similar to survival observed 60 years ago in developed countries. For example, in England and Wales, women diagnosed with breast cancer in 1945–1949 had an estimated overall RS of 44%, and it was 55% in Connecticut, USA.^19^ In 1950–1954, 5‐year RS was 48% in England and Wales, 56% in Connecticut, 52% in Finland and 57% in Norway.^19^ Currently, the 5‐year ASRS from breast cancer is 81.8% in Europe (79.2% in UK and Ireland) for cases diagnosed in 1999–2007^14^ and 91.1% in the USA (83.1% in black women) for cases diagnosed in 2008–2014.^20^

Stage was an important predictor of survival, even after adjusting for age and country HDI. Of those with known stage, 64.9% were diagnosed at a late stage. An equally high proportion of late‐stage disease (77%) was described in a meta‐analysis including 24,213 women from a variety of hospital settings within SSA.^21^ A study on breast cancer stage at diagnosis using population‐based data in Abidjan, Cote d'Ivoire and Brazzaville, Congo for cases diagnosed from 2008 to 2009 reported 74 and 81% of breast cancers diagnosed at Stages III and IV.^22^ It has to be noted that there is a considerable delay between first symptoms and presentation to health care practitioner; a recent systematic review found between 3 to over 6 months delay, and there is an additional 3–6 months interval between first presentation to health care practitioner and confirmation of diagnosis of breast cancer in SSA^23^; while in comparison, the median time from first presentation at the health care setting to diagnosis in 2004–2005 was on average 25 days (range:14–44 days) in the Aarhus county of Denmark. The median overall time from first symptom recognition to diagnosis has been estimated at 7.9 months in Accra, Ghana,^24^ 8.5 months in Western Cape, South Africa, at more than 10 months in Abidjan, Cote d'Ivoire^25^ and at 15 months in rural Rwanda^26^; in contrast, in 2006, about 30% of all breast cancer cases in England, Scotland and Wales were diagnosed asymptomatically by screening.^27^

Some reasons for late presentation in SSA include low breast cancer awareness,^28^ difficult access to healthcare (both physical^29^ and economic), fear, distrust of conventional medicine and belief in alternative sources of healing.^23^, ^30^ Additionally, pathways within the healthcare system often hinder early diagnosis.^31^, ^32^ Unfavorable tumor biology such as triple‐negative disease or the luminal‐B‐like phenotype may also be associated with late stage presentation,^33^ as these tumors generally grow faster leading to late stage at diagnosis. Furthermore, the possibility of underestimation of Stage IV disease due to the paucity of facilities for accurate staging needs to be considered in SSA. This can also explain variations in proportion of Stage IV disease between registries depending on local availability and practice.

In addition to diagnostic delays, there are further delays between confirmation of diagnosis and onset and completion of therapy, as a result of both patient‐ and system‐related factors. However, even for patients with the same stage at diagnosis, those in a high HDI country had a better survival experience. This may be linked to the availability and access to treatment as most people in low‐ and middle‐income countries (LMIC) have to pay out‐of‐pocket for healthcare.^34^ Hence improving survival among women diagnosed with breast cancers in SSA would require at least two major developments that are downstaging (through improved breast health awareness and clinical breast examination) and improved access to diagnosis and adequate treatment.^35^

Age was not an important predictor of survival after adjusting for the effect of stage and country HDI in our study. In most developed countries, poorer survival at 5 years is observed among older women^14^ particularly in recent years^36^ and also among women diagnosed with early‐onset breast cancer, before the age of 40.^37^ This has been linked to the effect of screening in the middle‐age group^36^ resulting in a lead‐time bias, and less aggressive treatment in older women.^38^ However, in Africa, the population is young, and most of the patients are diagnosed before age 55. Young age at diagnosis has been associated with higher proportions of familial breast cancer with BRCA mutations but also unfavorable tumor biology factors. Since there may be a lack of the large group of middle‐aged breast cancer patients with favorable tumor biology seen elsewhere, this could be one of the reasons why no difference in survival was detected.

There are several well‐known potential sources of bias in survival data—particularly from cancer registries in low‐income settings, which should be considered in interpreting these results.

First, although all participating registries were population‐based, the level of completeness of ascertainment of incident breast cancer cases is not known. Although all registries, as members of AFRCN, are evaluated as registering 70% or more of the incident cancers in their population,^39^ only five (Eastern Cape, Harare, Kampala, Nairobi and Seychelles) were of a quality permitting their publication in Cancer Incidence in Five Continents for the relevant period.^40^ Of course, this is only a source of bias if the cases missed by registration are nonrandom, with respect to their prognosis. Since few of the registries have access to (or use) death certificates from vital registration as a source of information, one might suppose that there was differential loss of fatal cases. On the other hand, inclusion of cases notified to the registry *via* death registration, which would otherwise have been missed (so‐called Death Certificate Initiated “DCI” cases), is known to bias survival in the opposite direction.^41^

Only one registry (Mauritius) relied entirely on passive follow‐up (linkage with death certificates) to ascertain vital status and identify cases that had died. This method potentially biases survival upwards, if there is a failure of record linkage, or cancer cases have migrated out of the registry area before dying. However, we actively followed up a 10% random sample of the breast cancer cases who were alive as per passive follow‐up—none of them had in fact died.

With active follow up, despite all attempts to trace cancer patients, a varying proportion is lost to follow up before the closing date of the study. Again, this is only a problem if these cases are more, or less, likely to have died compared to those that were successfully followed up. In Abidjan, cases with advanced breast cancer were more likely to be LFU by Year 3, but for the rest of the registries, among patients with known stage, LFU was non‐differential by either stage or age at diagnosis.

However, there was a large proportion of patients with missing information on stage at diagnosis (53%), in spite of active follow‐up done by registry staff. The proportion of patients with unknown stage at diagnoses varied from 10% in Seychelles to 74% in Kyadondo, Kampala. Some of the possible reasons for the absence of stage information are challenges in record keeping and inadequate resources to adequately stage patients (limited access and availability of financial resources to pay for these investigations as well as a limited plateau technique in some areas, lacking ultrasounds, X‐rays and CT‐scans). In high‐income countries, it has been shown that older patients are less likely to have exhaustive investigations compared to younger women,^42^ however, in our context, most of our patients are below age 55.

To better understand the pattern of missing stage data, using data from four registries: Bamako–Mali, Bulawayo–Zimbabwe, Benin–Cotonou and Nairobi–Kenya, we compared the available information on stage to data that was updated following active record finding for 90 patients: We found a similar distribution of early and late stage disease among patients in both groups.

With respect to generalizing the results to the populations studied, the size of the sampling fraction is relevant, thus with larger uncertainty intervals for some registries.

In order to estimate the RS, we used national life tables to obtain the “expected” rates of death. RS estimates above 100% were observed in some age groups (Supporting Information Table S2), indicating higher survival in this cohort of women coming from urban areas compared to that observed in the general population of women of the same age in the same country using country‐specific life tables inclusive of both rural and urban areas. Also, we categorized the registries according to the country‐level HDI, however, the level of development of an urban capital will be different from that of a rural setting. The countries with medium HDI represented were South Africa, Namibia and Kenya. However, the majority of the patients in this category were from Eastern Cape, South Africa, which is a rural area, with a level of development lower than that of South Africa as a whole. This could explain why little difference was observed between countries with medium and low HDI. However, correlations have been shown between national breast cancer mortality‐to‐incidence ratio (MIR) and country HDI with the use of GLOBOCAN 2012.^43^, ^44^ Finally, we have not adjusted for other important predictors of survival like treatment received and tumor biology.

Despite the limitations, this work produces estimates of survival by stage, not previously estimated from SSA using population‐level data. Due to the absence of mortality data, modelled survival was used to make estimates for mortality from most SSA countries in GLOBOCAN 2012.^45^ This work produces survival estimates from actual data from more registries, which could be used to inform public health authorities on breast cancer survival in Africa and be used to improve models for breast cancer mortality from SSA.

## Supporting information


**Table S1** Registries with potential for 5‐year follow‐up time
**Table S2**: Age‐specific relative survival and age‐standardized relative survival (ASRS) by registry
**Table S3**: Relative survival (RS) by stage at diagnosis and registry
**Figure S1**: Age distribution at diagnosis of breast cancer cases by registry
**Figure S2**: Stage distribution by registry
**Figure S3**: Overall survival for breast cancer by registry.

## References

[1] Parkin DM , Bray F , Ferlay J , et al. Cancer in Africa 2012. Cancer Epidemiol Biomarkers Prev 2014;23:953–66. 24700176 10.1158/1055-9965.EPI-14-0281

[2] Allemani C , Weir HK , Carreira H , et al. Global surveillance of cancer survival 1995–2009: analysis of individual data for 25 676 887 patients from 279 population‐based registries in 67 countries (CONCORD‐2). Lancet 2015;385:977–1010. 25467588 10.1016/S0140-6736(14)62038-9 PMC4588097

[3] Sankaranarayanan R, Swaminathan R, Lucas E, eds. Cancer survival in Africa, Asia, the Caribbean and Central America. Lyon: International Agency for Research on Cancer, World Health Organization, 2011 Available from: http://survcan.iarc.fr/survivalcitation.php.

[4] Allemani C , Matsuda T , Di Carlo V , et al. Global surveillance of trends in cancer survival 2000–14 (CONCORD‐3): analysis of individual records for 37 513 025 patients diagnosed with one of 18 cancers from 322 population‐based registries in 71 countries. Lancet 2018;391:1023–75. 29395269 10.1016/S0140-6736(17)33326-3 PMC5879496

[5] Brenner H , Swaminathan R . Statistical methods for cancer survival analysis In: Sankaranarayanan R, Swaminathan R, Lucas E, eds Cancer survival in Africa, Asia, the Caribbean and Central America (SurvCan). IARC scientific publications, vol. 162 Lyon: International Agency for Research on Cancer, World Health Organization, 2011 [cited 2018 Mar 16]; Available from: http://survcan.iarc.fr/survival/chap2.pdf.

[6] Dickman PW , Coviello E . Estimating and modeling relative survival. Stata J 2015;15:186–215.

[7] Ederer F , Axtell LM , Cutler SJ . The relative survival rate: a statistical methodology. Natl Cancer Inst Monogr 1961;6:101–21. 13889176

[8] Corazziari I , Quinn M , Capocaccia R . Standard cancer patient population for age standardising survival ratios. Eur J Cancer 2004;40:2307–16. 15454257 10.1016/j.ejca.2004.07.002

[9] WHO . By category | Life tables. Geneva, Switzerland: WHO [cited 2017 Dec 22]; Available from http://apps.who.int/gho/data/node.main.687?lang=en.

[10] Sobin LH, Gospodarowicz MK, Wittekind C, eds. TNM classification of malignant tumors, 7th edn. Hoboken, NJ: Wiley, 2011.

[11] Greene F , Page D , Fleming I , et al. Breast In: Amin MB, Edge S, Greene F, et al., eds AJCC cancer staging manual, 8th edn. New York: Springer, 2017 589–636.

[12] United Nations Development Programme . Human Development Index (HDI). Human Development Reports. New York, NY: UNDP, 2018 [cited 2018 Aug 10]; Available from: http://hdr.undp.org/en/content/human-development-index-hdi.

[13] United Nations Development Programme . Human development report 2016. New York, NY: UNDP, 2016 193.

[14] De Angelis R , Sant M , Coleman MP , et al. Cancer survival in Europe 1999‐2007 by country and age: results of EUROCARE‐5 ‐ a population‐based study. Lancet Oncol 2014;15:23–34. 24314615 10.1016/S1470-2045(13)70546-1

[15] Ries LAG, Young JL, Keel GE, et al., eds. SEER Survival Monograph: Cancer Survival Among Adults: U.S. SEER Program, 1988‐2001, NIH Pub. No. 07‐6215. Bethesda, MD: National Cancer Institute, SEER Program, 2007 Available from: http://www.seer.cancer.gov.

[16] Australian Institute of Health and Welfare . Cancer survival and prevalence in Australia: period estimates from 1982 to 2010. Asia Pac J Clin Oncol 2013;9:29–39. 23418847 10.1111/ajco.12062

[17] Gondos A , Chokunonga E , Brenner H , et al. Cancer survival in a southern African urban population. Int J Cancer 2004;112:860–4. 15386382 10.1002/ijc.20471

[18] Gondos A , Brenner H , Wabinga H , et al. Cancer survival in Kampala, Uganda. Br J Cancer 2005;92:1808–12. 10.1038/sj.bjc.6602540. 15827554 PMC2362045

[19] Cutler SJ . International Symposium on end results of cancer therapy. Survival tables. Natl Cancer Inst Monogr 1964;15:387–446. 14255801

[20] Noone AM, Howlader N, Krapcho M, et al., eds. SEER Cancer Statistics Review, 1975–2015. Bethesda, MD: National Cancer Institute, 2016 Available from: https://seer.cancer.gov/csr/1975_2015/.

[21] Jedy‐Agba E , Mccormack V , Adebamowo C , et al. Stage at diagnosis of breast cancer in sub‐Saharan Africa: a systematic review and meta‐analysis. Lancet Glob Health 2016;4:e923–35. 27855871 10.1016/S2214-109X(16)30259-5 PMC5708541

[22] Islami F , Lortet‐Tieulent J , Okello C , et al. Tumor size and stage of breast cancer in Côte d'Ivoire and Republic of Congo—results from population‐based cancer registries. Breast 2015;24:713–7. 26371692 10.1016/j.breast.2015.08.011

[23] Espina C , McKenzie F , dos‐Santos‐Silva I . Delayed presentation and diagnosis of breast cancer in African women: a systematic review. Ann Epidemiol 2017;27:659–671.e7. 29128086 10.1016/j.annepidem.2017.09.007 PMC5697496

[24] Clegg‐Lamptey J , Dakubo J , Attobra YN . Why do breast cancer patients report late or abscond during treatment in Ghana? A pilot study. Ghana Med J 2009;43:127–31. 20126325 PMC2810246

[25] Toure M , Nguessan E , Bambara AT , et al. Facteurs liés au diagnostic tardif des cancers du sein en Afrique‐sub‐saharienne: Cas de la Côte d'Ivoire. Gynecol Obstet Fertil 2013;41:696–700. 24210776 10.1016/j.gyobfe.2013.08.019

[26] Pace LE , Mpunga T , Hategekimana V , et al. Delays in breast cancer presentation and diagnosis at two rural cancer referral centers in Rwanda. Oncologist 2015;20:780–8. 26032138 10.1634/theoncologist.2014-0493 PMC4492236

[27] Cheung S , Greenway N , Lagord C , et al. *A UK analysis of all symptomatic and screen‐detected breast cancers in 2006* All Breast Cancer Report. NHS Cancer Screening Programmes and NCIN; 2009.

[28] Akuoko CP , Armah E , Sarpong T , et al. Barriers to early presentation and diagnosis of breast cancer among African women living in sub‐Saharan Africa. PLoS One 2017;12:e0171024. 28192444 10.1371/journal.pone.0171024 PMC5305236

[29] Dickens C , Joffe M , Jacobson J , et al. Stage at breast cancer diagnosis and distance from diagnostic hospital in a periurban setting: a South African public hospital case series of over 1,000 women. Int J Cancer 2014;135:2173–82. 24658866 10.1002/ijc.28861 PMC4134722

[30] Donkor A , Wiafe S , Yarney J , et al. Factors contributing to late presentation of breast cancer in Africa: a systematic literature review. iMedPub 2015;8:1–10.

[31] Grosse Frie K , Samoura H , Diop S , et al. Why do women with breast cancer get diagnosed and treated late in sub‐Saharan Africa? Perspectives from women and patients in Bamako, Mali. Breast Care 2018;13:39–43. 29950966 10.1159/000481087 PMC6016059

[32] McKenzie F , Zietsman A , Galukande M , et al. Drivers of advanced stage at breast cancer diagnosis in the multicountry African breast cancer—disparities in outcomes (ABC‐DO) study. Int J Cancer 2018;142:1568–79. 29197068 10.1002/ijc.31187 PMC5838525

[33] Joffe M , Ayeni O , Norris SA , et al. Barriers to early presentation of breast cancer among women in Soweto, South Africa. PLoS One 2018;13:e0192071. 29394271 10.1371/journal.pone.0192071 PMC5796726

[34] Knaul F , Horton S , Yerramilli P , et al. Financing cancer care in low‐resource settings [Chapter 17] In: Gelband H, Jha P, Sankaranarayanan R, et al., eds Cancer: Disease Control Priorities, vol. 3, 3rd edn. Washington, DC: The International Bank for Reconstruction and Development/The World Bank, 2015. 26913340

[35] Anderson BO , Cazap E , El Saghir NS , et al. Optimisation of breast cancer management in low‐resource and middle‐resource countries: executive summary of the breast health global initiative consensus, 2010. Lancet Oncol 2011;12:387–98. 21463833 10.1016/S1470-2045(11)70031-6

[36] Quaglia A , Tavilla A , Shack L , et al. The cancer survival gap between elderly and middle‐aged patients in Europe is widening. Eur J Cancer 2009;45:1006–16. 19121578 10.1016/j.ejca.2008.11.028

[37] Narod SA . Breast cancer in young women. Nat Rev Clin Oncol 2012;9:460–70. 22733233 10.1038/nrclinonc.2012.102

[38] Lavelle K , Todd C , Moran A , et al. Non‐standard management of breast cancer increases with age in the UK: a population based cohort of women ⩾65 years. Br J Cancer 2007;96:1197–203. 17387342 10.1038/sj.bjc.6603709 PMC2360138

[39] African Cancer Registry Network Membership Criteria . [cited 2018 Oct 25]; Available from: http://www.afcrn.org/index.php/membership/membership-criteria2.

[40] Bray F , Colombet M , Mery L , et al. Cancer incidence in five continents, vol. XI Lyon: International Agency for Research on Cancer, 2017 [cited 2018 Feb 21]; Available from: http://ci5.iarc.fr/CI5-XI/Default.aspx.

[41] Silcocks P . Survival of death certificate initiated registrations: selection bias, incomplete trace‐back or higher mortality? Br J Cancer 2006;95:1576–8. 17117185 10.1038/sj.bjc.6603418 PMC2360747

[42] Di Girolamo C , Walters S , Benitez Majano S , et al. Characteristics of patients with missing information on stage: a population‐based study of patients diagnosed with colon, lung or breast cancer in England in 2013. BMC Cancer 2018;18:492. 29716543 10.1186/s12885-018-4417-3 PMC5930770

[43] Hu K , Lou L , Tian W , et al. The outcome of breast cancer is associated with National Human Development Index and health system attainment. PLoS One 2016;11:e0158951. 27391077 10.1371/journal.pone.0158951 PMC4938431

[44] Bray F , Jemal A , Grey N , et al. Global cancer transitions according to the human development index (2008–2030): a population‐based study. Lancet Oncol 2012;13:790–801. 22658655 10.1016/S1470-2045(12)70211-5

[45] Ferlay J , Soerjomataram I , Dikshit R , et al. Cancer incidence and mortality worldwide: sources, methods and major patterns in GLOBOCAN 2012. Int J Cancer 2015;136:E359–86. 25220842 10.1002/ijc.29210

