# Supplementary material for: Breast cancer survival in sub‐Saharan Africa by age, stage at diagnosis and human development index: A population‐based registry study
Source: Int J Cancer. 2019 Jun 14;146(5):1208–18. doi: 10.1002/ijc.32406 (PMC7079125; doi:10.1002/ijc.32406)
Supplement: Supplementary file 1 — Table S1 Registries with potential for 5‐year follow‐up time Table S2: Age‐specific relative survival and age‐standardized relative survival (ASRS) by registry Table S3: Relative survival (RS) by stage at diagnosis and registry Figure S1: Age distribution at diagnosis of breast cancer cases by registry Figure S2: Stage distribution by registry Figure S3: Overall survival for breast cancer by registry. [file IJC-146-1208-s001.doc]

# APPENDIX TABLES

Appendix Table 1: Registries with potential for 5-year follow-up time

| **Country, Registry** | **Period of diagnosis** | **Number of cases included** | **Number of cases with potential for complete 5-year follow-up** | **Complete 5-year follow-up** | |
| --- | --- | --- | --- | --- | --- |
|  |  |  |  | Known Alive/Dead | % |
| Kenya, Eldoret | 2009 - 2013 | 78 | 48 | 27 | 56.3 |
| Kenya, Nairobi | 2009 - 2013 | 141 | 95 | 59 | 62.1 |
| Mauritius | 2005 - 2009 | 491 | 380 | 380 | 100 |
| Namibia | 2012 - 2013 | 64 | 31 | 26 | 83.9 |
| South Africa, Eastern Cape | 2008 - 2013 | 313 | 210 | 145 | 69.0 |
| Seychelles | 2008 – 2013 | 105 | 82 | 72 | 87.8 |
| Uganda, Kyadondo | 2009 – 2013 | 112 | 96 | 58 | 60.4 |
| Zimbabwe, Harare | 2009 – 2013 | 174 | 120 | 117 | 97.5 |

Appendix Table 2: Age-specific relative survival and age-standardised relative survival (ASRS) by registry

| **Country, Registry** | **1-year relative survival** | | | | | | | | | | **1-year ASRS** | |
| --- | --- | --- | --- | --- | --- | --- | --- | --- | --- | --- | --- | --- |
|  | **< 45** | **95% CI** | **45 - 54** | **95% CI** | **55 - 64** | **95% CI** | **65-74** | **95% CI** | **All ages** | **95% CI** | **All ages** | **95% CI** |
| Benin, Cotonou | 87.3 | 69.1 – 95.3 | 81.2 | 56.5 – 93.0 | 84.8 | 49.0 – 97.2 | 104.8 | - | 83 | 69.6 – 91.1 | 100 | - |
| Cote d’Ivoire, Abidjan | 88.5 | 79.5 – 94.0 | 84.4 | 71.1 – 92.3 | 90.2 | 68.5 – 98.6 | 85.3 | 59.4 – 97.7 | 87 | 80.8 -91.6 | 94.3 | 83.4 – 98.1 |
| Ethiopia, Addis | 90.8 | 85.6 – 94.2 | 98.4 | 91.1 – 100.3 | 89.3 | 76.2 – 95.9 | 89.7 | 66.4 – 98.9 | 92.2 | 88.6 – 94.8 | 92.5 | 81.4 – 97.1 |
| Kenya, Eldoret | 84.7 | 66.2 – 93.9 | 87.2 | 64.1 – 96.3 | 89 | 39.3 – 99.8 | 103.3 | - | 85.3 | 72.9 – 92.7 | 88.3 | 38.2 – 98.4 |
| Kenya, Nairobi | 96.6 | 84.5 – 99.9 | 93.7 | 80.0 – 98.6 | 94.2 | 75.1 – 99.7 | 103.6 | - | 95.9 | 89.9 – 98.8 | 103.8 | - |
| Mali, Bamako | 70.4 | 45.3 – 85.8 | 81.7 | 43.3 – 95.8 | 102.2 | - | 45.2 | 10.3 – 77.4 | 72.5 | 55.9 – 84.0 | 72.3 | 55.7 – 83.6 |
| Mauritius | 94.4 | 87.7 – 97.6 | 90.8 | 84.3 – 94.8 | 90.9 | 84.1 – 95.1 | 84.1 | 71.9 – 91.8 | 91.6 | 88.4 – 94.0 | 91.3 | 86.0 – 94.7 |
| Mozambique, Maputo | 66.2 | 36.3 – 84.8 | 89.3 | 41.6 – 99.5 | 92.9 | 51.9 – 100.8 | 52.7 | 0.6 – 96.0 | 77.1 | 58.2 – 88.6 | 73.2 | 38.0 – 90.5 |
| Namibia | 94.3 | 62.8 – 99.9 | 95.7 | 68.8 – 100.3 | 101.5 |  | 89.3 | 34.8 – 102.0 | 97.1 | 87.2 – 100.5 | 99.8 |  |
| SA, Eastern Cape | 88.2 | 77.7 – 94.3 | 80.2 | 67.1 – 88.8 | 66.5 | 54.3 – 76.4 | 79.2 | 62.3 – 90.2 | 77 | 71.2 – 82.0 | 76 | 68.3 – 82.1 |
| Seychelles | 100.2 |  | 92.7 | 72.9 – 98.5 | 97.5 | 78.7 – 100.5 | 87.9 | 55.3 – 98.7 | 89.4 | 81.0 – 94.8 | 85 | 73.0 – 91.9 |
| Uganda, Kyadondo | 74.6 | 57.3 – 85.9 | 67.1 | 47.9 – 80.7 | 85 | 57.9 – 96.2 | 103.6 | - | 75.4 | 65.1 – 83.2 | 85.5 | 60.3 – 95.3 |
| Zimbabwe, Bulawayo | 63.6 | 23.4 – 87.6 | 41.5 | 16.2 – 65.7 | 79.3 | 37.2 – 95.8 | 75.2 | 38.3 – 93.3 | 63 | 46.0 – 76.3 | 75.2 | 51.4 – 88.5 |
| Zimbabwe, Harare | 75 | 61.1 – 85.0 | 75.5 | 60.7 – 85.7 | 70.1 | 51.6 – 83.0 | 72.9 | 46.9 – 88.7 | 76.5 | 69.0 – 82.6 | 84.8 | 75.8 – 90.7 |
|  | **3-year relative survival** | | | | | | | | | | **3-year ASRS** | |
| Benin, Cotonou | 54.2 | 29.8 – 73.6 | 58.6 | 29.6 – 79.8 | 59.8 | 20.5 – 86.8 | 118.5 | - | 55.9 | 39.4 – 70.1 | 58.9 | 49.3 – 67.3 |
| Cote d’Ivoire, Abidjan | 56.2 | 44.3 – 66.8 | 57 | 39.7 – 71.5 | 50.6 | 27.4 – 71.2 | 48.9 | 23.6 – 73.3 | 55 | 46.6 – 62.9 | 64.8 | 35.5 – 83.4 |
| Ethiopia, Addis | 69.1 | 61.6 – 75.5 | 67.4 | 54.9 – 77.4 | 53.5 | 37.6 – 67.4 | 63.8 | 37.4 – 83.8 | 66.8 | 61.2 – 72.0 | 69.2 | 54.9 – 79.8 |
| Kenya, Eldoret | 47.7 | 27.0 – 66.1 | 62.6 | 35.1 – 81.8 | 30.8 | 4.5 – 65.3 | 109.8 | - | 52.4 | 37.3 – 66.0 | 87.1 | 3.0 – 99.5 |
| Kenya, Nairobi | 77 | 58.5 – 88.7 | 74.3 | 54.2 – 87.2 | 78.8 | 55.0 – 92.4 | 111.4 | - | 79.5 | 69.1 – 87.2 | 112.9 |  |
| Mali, Bamako | 41.6 | 19.2 – 63.0 | 59.6 | 22.9 – 84.4 | 77.2 | 9.7 – 103.1 | 16.8 | 0.8 – 54.7 | 46.4 | 29.6 – 62.0 | 46 | 27.5 – 62.8 |
| Mauritius | 87 | 78.7 – 92.4 | 76.7 | 68.3 – 83.3 | 86 | 78.0 – 91.8 | 71.6 | 57.3 – 82.9 | 83.4 | 79.1 – 87.1 | 86 | 77.8 – 91.3 |
| Namibia | 88.7 | 56.9 – 99.0 | 75.5 | 48.6 – 90.7 | 90.9 | 59.1 – 101.2 | 78.4 | 24.3 – 104.3 | 84.5 | 70.6 – 93.5 | 87.7 | 26.8 – 98.7 |
| SA, Eastern Cape | 58.7 | 44.0 – 71.1 | 57.9 | 41.9 – 71.4 | 35.1 | 23.2 – 47.4 | 64.8 | 43.7 – 82.0 | 49.5 | 42.4 – 56.5 | 49.3 | 40.0 – 57.9 |
| Seychelles | 80.6 | 50.4 – 93.8 | 81.9 | 60.7 – 92.8 | 92.4 | 73.3 – 99.5 | 85.6 | 51.5 – 100.8 | 82.6 | 72.4 – 90.2 | 82 | 66.1 – 90.9 |
| Uganda, Kyadondo | 43.4 | 24.3 – 61.4 | 30.5 | 13.9 – 49.3 | 18.3 | 3.0 – 44.6 | 56 | 6.5 – 94.6 | 33.2 | 22.2 – 44.8 | 27.1 | 12.2 – 44.6 |
| Zimbabwe, Bulawayo | 16.6 | 8.0 – 52.2 | 10.7 | 0.7 – 37.5 | - | - | 46.9 | 10.9 – 80.4 | 21.6 | 8.2 – 39.8 |  |  |
| Zimbabwe, Harare | 53.1 | 38.4 – 66.3 | 53 | 37.7 – 66.5 | 54.8 | 36.2 – 70.7 | 45.2 | 21.8 – 67.8 | 56.7 | 48.2 – 64.6 | 72.1 | 57.5 – 82.5 |
|  | **5-year relative survival** | | | | | | | | | | **5-year ASRS** | |
| Kenya, Eldoret | 36.4 | 16.7 – 57.1 | 52.2 | 22.6 – 76.5 | 31.7 | 4.6 – 67.2 | - | - | 43.7 | 27.7 – 59.2 | - | - |
| Kenya, Nairobi | 74.7 | 55.5 – 87.6 | 70 | 47.6 – 85.3 | 68 | 40.8 – 86.9 | - | - | 69.5 | 57.0 – 79.7 | 27.1 | 23.1 – 35.2 |
| Mauritius | 85.5 | 76.8 – 91.3 | 71.9 | 62.9 – 79.2 | 84.1 | 75.3 – 90.8 | 72 | 56.3 – 85.1 | 83.2 | 78.4 – 87.4 | 93.7 | 75.5 – 98.5 |
| Namibia | 63.3 | 30.0 – 85.5 | 77.3 | 49.7 – 92.8 | 94.5 | 61.5 – 105.2 | 87.4 | 27.1 – 116.3 | 78.5 | 62.4 – 90.2 | 80 | 22.2 – 96.8 |
| SA, Eastern Cape | 46.4 | 31.1 – 60.7 | 42.2 | 25.4 – 58.6 | 29.8 | 18.1 – 42.9 | 67.1 | 43.3 – 87.4 | 39.2 | 31.5 – 46.9 | 38.2 | 28.7 – 47.6 |
| Seychelles | 81.1 | 50.7 – 94.4 | 69.4 | 46.6 – 84.5 | 80.4 | 56.4 – 93.9 | 72.7 | 36.4 – 96.3 | 70.2 | 58.0 – 80.6 | 66.3 | 49.2 – 78.8 |
| Uganda, Kyadondo | 20.7 | 6.4 – 41.1 | 14.3 | 3.3 – 33.3 | 9.5 | 6.0 – 34.9 | - | - | 12.1 | 4.4 – 24.2 | 5.3 | 1.9 – 11.3 |
| Zimbabwe, Harare | 40.6 | 26.2 – 55.2 | 52.5 | 36.9 – 66.7 | 57.1 | 37.7 – 73.7 | 41.3 | 17.6 – 66.5 | 51.2 | 42.2 – 59.9 | 60.8 | 38.4 – 77.3 |

Appendix Table 3: Relative survival (RS) by stage at diagnosis and registry

| **Country, registry** | **Number of cases included** | **Cases with known stage** | **Number with late stage disease** | **Proportion with late stage disease** | **1 YEAR RS** | | | **3 YEAR RS** | | | **5 YEAR RS** | | |
| --- | --- | --- | --- | --- | --- | --- | --- | --- | --- | --- | --- | --- | --- |
|  |  |  |  |  | **(95%CI)** | | | **(95%CI)** | | | **(95%CI)** | | |
|  |  |  |  |  | **Early Stage** | **Late Stage** | **Missing stage** | **Early Stage** | **Late Stage** | **Missing stage** | **Early Stage** | **Late Stage** | **Missing stage** |
| **Benin, Cotonou** | 91 | 59 | 44 | 74.6 | 74.1 | 80.4 | 90.2 | 60.4 | 48.5 | 64.7 | – | - | – |
|  |  |  |  |  | (29.0 – 93.5) | (59.7 – 91.8) | (64.0 – 98.3) | (19.0 – 86.9) | (25.7 – 69.2) | (35.6 – 84.1) |  |  |  |
| **Cote d’Ivoire, Abidjan** | 209 | 89 | 56 | 62.9 | 95.5 | 87.4 | 85.5 | 85 | 40.7 | 52.7 | – | - | – |
|  |  |  |  |  | (78.2 – 100.4) | (74.0 – 94.6) | (76.7 – 91.4) | (64.3 – 96.4) | (25.2 – 55.9) | (41.4 – 63.2) |  |  |  |
| **Ethiopia, Addis** | 389 | 128 | 69 | 53.9 | 94.1 | 90.8 | 92.5 | 71.4 | 57.5 | 68.6 | – |  | – |
|  |  |  |  |  | (83.3 – 98.4) | (80.3 – 96.3) | (88.0 – 95.5) | (56.8 – 82.2) | (43.7 – 69.4) | (61.6 – 74.8) |  |  |  |
| **Kenya, Eldoret** | 78 | 26 | 21 | 80.8 | 101.5^ǂ^ | 90.5 | 83.5 | 105.3^ǂ^ | 49.2 | 49 | 73.1^ǂ^ | 39 | 45.4 |
|  |  |  |  |  | (-) | (64.8 – 98.4) | (68.1 – 92.7) | (–) | (21.9 –72.4) | (30.3 – 66.0) | (5.9 – 103.6) | (13.1 – 65.7) | (26.4 – 63.6) |
| **Kenya, Nairobi** | 141 | 81 | 54 | 66.7 | 92.9 | 101.7 | 91.5 | 81 | 80.3 | 78.1 | 72 | 74.8 | 63.6 |
|  |  |  |  |  | (72.0 – 99.1) | (–) | (79.0 – 97.2) | (57.0 – 93.6) | (62.0 – 91.7) | (59.9 – 89.7) | (46.3 – 88.5) | (53.4 - 89.7) | (42.5 – 89.7) |
| **Mali, Bamako** | 48 | 36 | 30 | 83.3 | 83.4^ǂ^ | 65.4 | 91.6 | 86.5^ǂ^ | 25.3 | 82.1 | – | - | – |
|  |  |  |  |  | (24.4 – 99.1) | (44.5 – 80.3) | (49.7 – 99.9) | (25.3 – 102.8) | (9.7 – 44.8) | (38.7 – 98.7) |  |  |  |
| **Mauritius** | 491 |  |  |  | – | – | – | – | – | – | – |  | – |
| **Mozambique, Maputo** | 42 | 28 | 16 | 57.1 | 81 | 81.8 | 73.7 | – | – | – | – | - | – |
|  |  |  |  |  | (39.8 – 95.3) | (52.0 – 94.7) | (37.6 – 91.5) |  |  |  |  |  |  |
| **Namibia** | 64 | 54 | 31 | 57.4 | 101.9 | 95.8 | 91.4 | 89.4 | 81.3 | 83.9 | 79.7 | 74.5 | 89.1 |
|  |  |  |  |  | (-) | (77.9 – 100.9) | (46.4 – 100.6) | (63.5 -99.8) | (59.4 – 94.5) | (40.0 – 100.8) | (50.3 – 96.2) | (50.2 – 91.2) | (42.5 – 107.1) |
| **SA, Eastern Cape** | 313 | 106 | 66 | 62.3 | 92.4 | 74.7 | 75.9 | 60.3 | 39.4 | 51.9 | 44.2 | 29.3 | 42.7 |
|  |  |  |  |  | (76.7 – 99.0) | (61.4 – 84.4) | (68.6 – 81.9) | (39.1 – 77.6) | (25.7 – 53.3) | (42.8 – 60.4) | (23.4 – 64.8) | (16.4 – 44.1) | (32.7 – 52.7) |
| **Seychelles** | 105 | 94 | 40 | 42.6 | 99 | 79.9 | 78.1 | 95 | 68.7 | 74.1 | 84.7 | 53.6 | 61.2 |
|  |  |  |  |  | (88.4 – 101.8) | (63.1 – 90.3) | (39.8 – 97.0) | (81.5-102.1) | (50.9 – 82.0) | (34.6 – 98.4) | (67.4 – 96.9) | 35.1 – 69.8) | (20.2 – 94.1) |
| **Uganda, Kyadondo** | 112 | 29 | 20 | 69 | 66^ǂ^ | 86.3 | 73.3 | 41.1^ǂ^ | 37.9 | 29.9 | 42.1^ǂ^ | 30.4 | 3.7 |
|  |  |  |  |  | (26.1 – 88.8) | (60.7 – 96.7) | (60.8 - 82.5) | (9.9 – 72.8) | (15.3 – 61.3) | (17.1 – 44.2) | (10.1 – 74.4) | (9.7 – 55.6) | (0.3 – 15.0) |
| **Zimbabwe, Bulawayo** | 54 | 42 | 31 | 73.8 | 103.2 | 57.2 | 44.7 | 54.8^ǂ^ | 18.6 | – | – | - | – |
|  |  |  |  |  | (–) | (36.1 – 74.1) | (11.5 – 75.4) | (6.3 – 92.6) | (4.7 – 40.7) |  |  |  |  |
| **Zimbabwe, Harare** | 174 | 84 | 77 | 91.7 | 87.9^ǂ^ | 74.9 | 76.8 | 77.2^ǂ^ | 46.6 | 63.6 | 81.6^ǂ^ | 37.6 | 60.2 |
|  |  |  |  |  | (34.3 – 100.4) | (63.2 – 83.7) | (66.0 – 84.9) | (27.9 – 99.4) | (34.3 – 58.4) | (51.5 – 74.1) | (29.5 – 105.1) | (25.3 – 50.4) | (47.4 – 71.9) |
| **All cases** | 2311 | 844 | 547 | 64.8 | 93.5 | 82 | 83 | 78 | 51.1 | 59 | 69.3 | 40.3 | 49.2 |
|  |  |  |  |  | (89.4 - 96.3) | (78.1 - 85.3) | (80.1 - 85.6) | (71.6 - 83.3) | (46.0 - 56.0) | (55.1 - 62.8) | (61.5 - 76.2) | (34.9 - 45.7) | (44.8-53.6) |

^ǂ^ *Less than 10 cases available for survival analyses* *in category*

# APPENDIX FIGURES


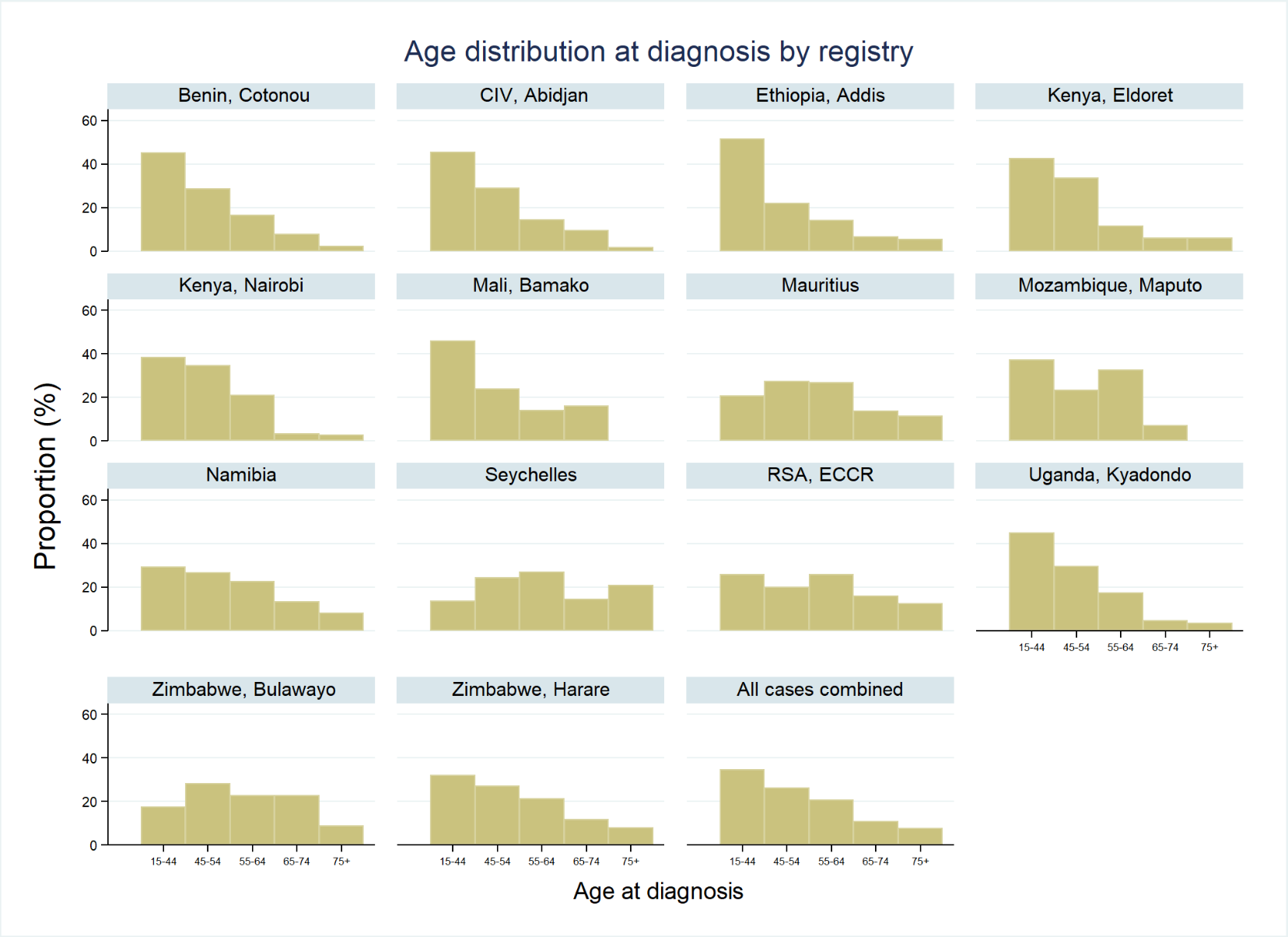


Appendix Figure 1: Age distribution at diagnosis of breast cancer cases by registry


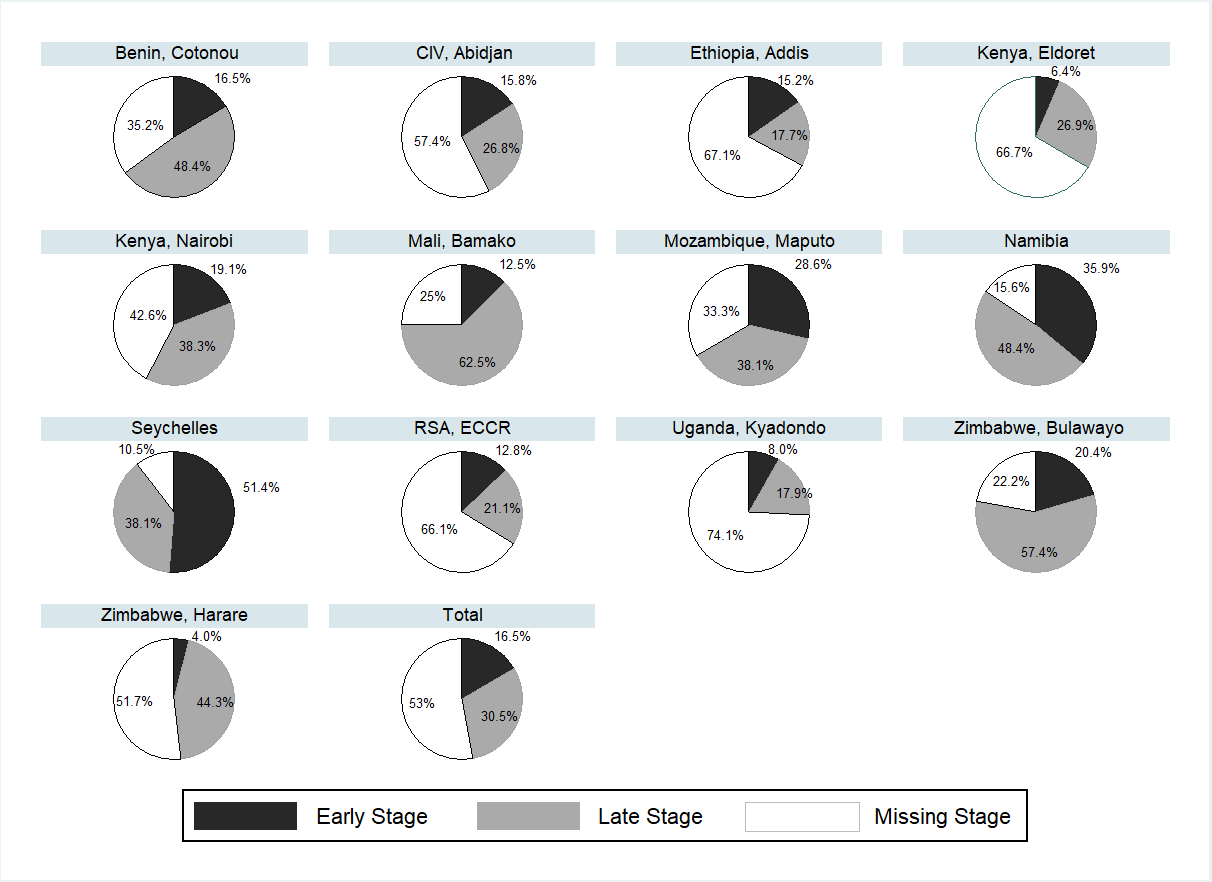


Appendix Figure 2: Stage distribution by registry


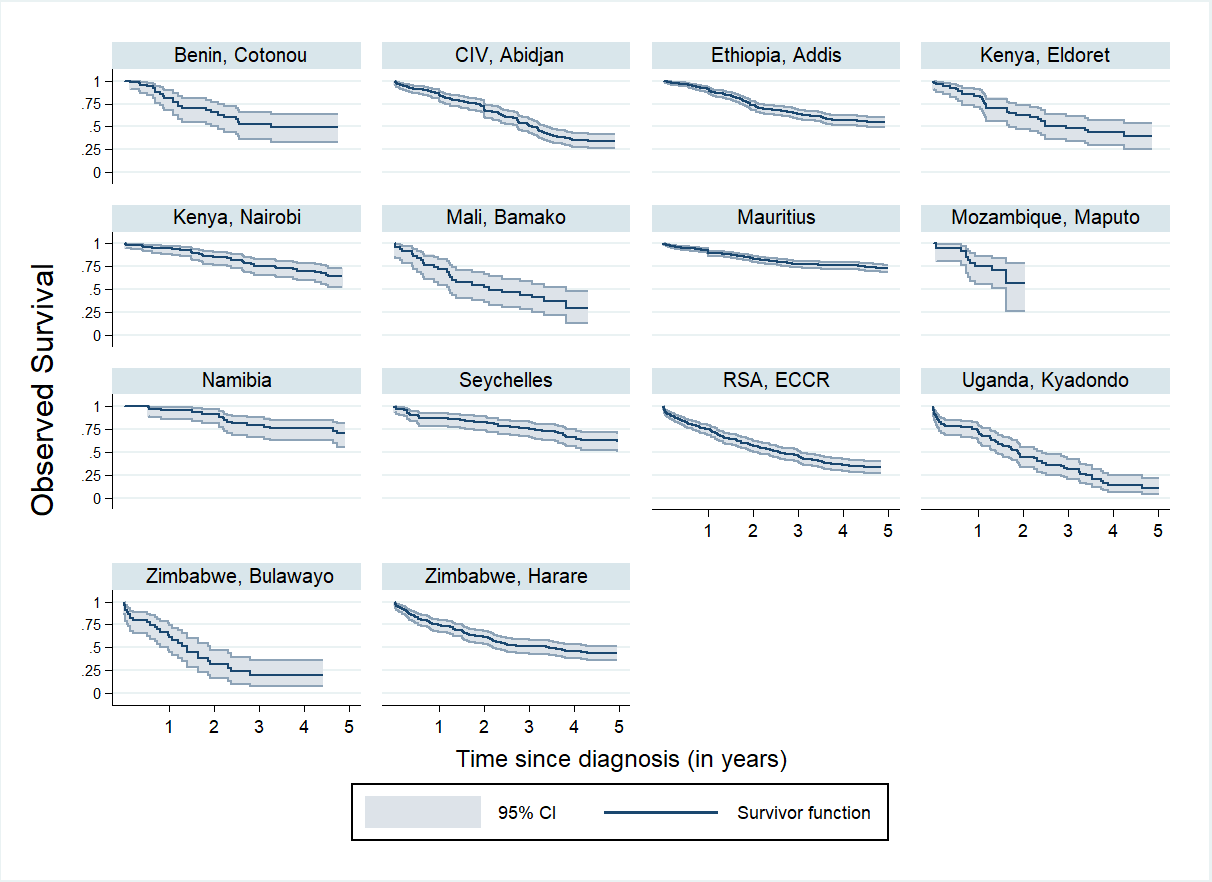


Appendix Figure 3: Overall survival for breast cancer by registry

**Supplementary Information:**

**Modelling of lifetables:**

Single year and 5-year-age abridged lifetables for the years 2000-2016 at national level was retrieved from the WHO Global Health Observatory. We obtained age-specific death rates, calculated from information on deaths among persons in the age group at age *x* during a given time period and the total person-years for the population in the same time period. A full description of the methods is available elsewhere <https://www.who.int/healthinfo/statistics/LT_method.pdf?ua=1>.

The number of deaths and person-time by sex, year and country were used to estimate mortality rates using a Poisson regression and a flexible function to expand the abridged age groups (0-4, 5-9, 10-14 …80+) to single ages (0,1,2,3,4,5...99). Briefly, we used the number of deaths and person-time by year and sex for each country separately. Smoothed age-specific mortality rates were derived using Poisson regression modelling by piecewise and spline function using eight knots with locations at ages 0-10 (three knots), 15-30 (three knots) and 50-85+ (two knots). The method chosen was fully described and explored by Rachet and colleagues (2015) (<https://bmcpublichealth.biomedcentral.com/track/pdf/10.1186/s12889-015-2534-3>).
